# Bioactivation mechanisms of *N*‐hydroxyaristolactams: Nitroreduction metabolites of aristolochic acids

**DOI:** 10.1002/em.22321

**Published:** 2019-08-16

**Authors:** Yoshiharu Okuno, Radha Bonala, Sivaprasad Attaluri, Francis Johnson, Arthur P. Grollman, Viktoriya S. Sidorenko, Yoshimitsu Oda

**Affiliations:** ^1^ Department of Applied Chemistry and Biochemistry, National Institute of Technology Wakayama College 77 Noshima, Nada, Gobo‐shi, Wakayama 644‐0023 Japan; ^2^ Department of Material Science and Engineering, Material Science and Engineering Wakayama National College of Technology, Gobo‐shi Wakayama 644‐0023 Japan; ^3^ Department of Pharmacological Sciences Stony Brook University Stony Brook New York 11794 USA; ^4^ Department of Chemistry Stony Brook University Stony Brook New York 11794 USA; ^5^ Department of Medicine Stony Brook University Stony Brook New York 11794 USA; ^6^ Institute of Life and Environmental Sciences Osaka Shin‐Ai College 6‐2‐28 Tsurumi, Tsurumi‐ku, Osaka 538‐0053 Japan

**Keywords:** *umu* test, *N,O*‐acetyltransfer, sulfonation, aristolactam‐DNA adducts, HK‐2 cytosols

## Abstract

Aristolochic acids (AAs) are human nephrotoxins and carcinogens found in concoctions of *Aristolochia* plants used in traditional medicinal practices worldwide. Genotoxicity of AAs is associated with the formation of active species catalyzed by metabolic enzymes, the full repertoire of which is unknown. Recently, we provided evidence that sulfonation is important for bioactivation of AAs. Here, we employ *Salmonella typhimurium umu* tester strains expressing human *N*‐acetyltransferases (NATs) and sulfotransferases (SULTs), to study the role of conjugation reactions in the genotoxicities of *N*‐hydroxyaristolactams (AL‐I‐NOH and AL‐II‐NOH), metabolites of AA‐I and AA‐II. Both *N*‐hydroxyaristolactams show stronger genotoxic effects in *umu* strains expressing human NAT1 and NAT2, than in the parent strain. Additionally, AL‐I‐NOH displays increased genotoxicity in strains expressing human SULT1A1 and SULT1A2, whereas AL‐II‐NOH shows enhanced genotoxicity in SULT1A1/2 and SULT1A3 strains. 2,6‐Dichloro‐4‐nitrophenol, SULTs inhibitor, reduced *umuC* gene expression induced by *N*‐hydroxyaristolactams in SULT1A2 strain. *N*‐hydroxyaristolactams are also mutagenic in parent strains, suggesting that an additional mechanism(s) may contribute to their genotoxicities. Accordingly, using putative SULT substrates and inhibitors, we found that cytosols obtained from human kidney HK‐2 cells activate *N*‐hydroxyaristolactams in aristolactam‐DNA adducts with the limited involvement of SULTs. Removal of low‐molecular‐weight reactants in the 3.5–10 kDa range inhibits the formation of aristolactam‐DNA by 500‐fold, which could not be prevented by the addition of cofactors for SULTs and NATs. In conclusion, our results demonstrate that the genotoxicities of *N*‐hydroxyaristolactams depend on the cell type and involve not only sulfonation but also *N*,O‐acetyltransfer and an additional yet unknown mechanism(s). Environ. Mol. Mutagen. 2019. © 2019 Wiley Periodicals, Inc.

**Abbreviations**
AA‐I: aristolochic acid I or 8‐methoxy‐6‐nitrophenanthro‐[3,4‐d]‐1,3‐dioxole‐5‐carboxylic acid
AA‐II: aristolochic acid II or 6‐nitrophenanthro‐[3,4‐d]‐1,3‐dioxole‐5‐carboxylic acid
AAN: aristolochic acid nephropathy
AAs or AA: collective terms for various aristolochic acids
AL‐DNA: aristolactam‐DNA adduct
AL‐I: aristolactam I
AL‐II: aristolactam II
AL‐II‐NOH: *N*‐hydroxyaristolactam II
AL‐II‐N‐OSO_3_H: aristolactam‐II‐N‐sulfate or *N*‐sulfonyloxyaristolactam II
AL‐I‐NOH: *N‐*hydroxyaristolactam I
AL‐I‐N‐OSO_3_H: aristolactam‐I‐N‐sulfate or *N*‐sulfonyloxyaristolactam I
BEN: Balkan endemic nephropathy
CYP1A2/1: cytochrome P450 1A1 and cytochrome P450 1A2
DMSO: dimethyl sulfoxide
DNCP: 2,6‐dichloro‐4‐nitrophenol
FAD: flavin adenine dinucleotide
FMN: flavin mononucleotide
HK‐2: human kidney cell line
NADH: nicotinamide adenine dinucleotide
NADPH: nicotinamide adenine dinucleotide phosphate
NAT: N‐acetyltransferase
NQO1: NAD(P)H:quinone oxidoreductase 1
NR: nitroreduction
*O*‐AT: bacterial O‐acetyltransferase
PAGE: polyacrylamide gel electrophoresis
PAP: 3′‐phosphoadenosine‐5′‐phosphate
PAPS: 3′‐phosphoadenosine‐5′‐phosphosulfate
PCP: pentachlorophenol
*Salmonella thyphimurium*: *S. thyphimurium*
ssDNA: salmon sperm DNA
SULT: sulfotransferase.

## INTRODUCTION

**Figure 1:**
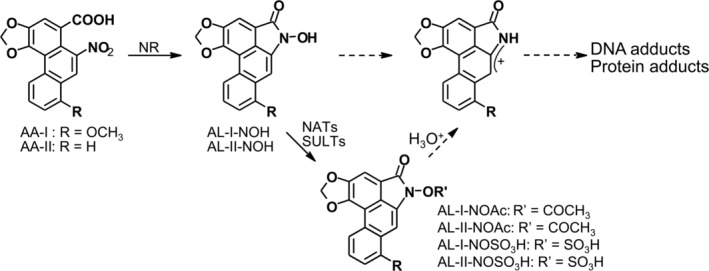
Proposed pathways for the metabolic activation of the aristolochic acids. Four‐electron NR of AA‐I and AA‐II produces their respective *N*‐hydroxyaristolactams, AL‐I‐NOH and AL‐II‐NOH. *N*‐hydroxyaristolactams decompose to form electrophilic cyclic nitrenium/carbenium ions with delocalized positive charge, or undergo conjugation reactions catalyzed by sulfotransferases (SULTs) and N‐acetyltransferases (NATs). Resulting *N*‐sulfonyloxyaristolactams (AL‐I‐NOSO_3_H and AL‐II‐NOSO_3_H) and *N*‐acetoxylaristolactams (AL‐I‐NOAc and AL‐II‐NOAc) readily undergo solvolysis, forming the same active species as *N*‐hydroxyaristolactams. *N*‐hydroxyaristolactams are more stable than their esters and lead to DNA adduction at much slower rates.

*Aristolochia* botanicals have been used since antiquity to treat a variety of human conditions (Dawson 1927; Grollman and Marcus 2016). These herbs contain a family of structurally related nitrophenanthrene carboxylic acids, of which aristolochic acid I (AA‐I) and aristolochic acid II (AA‐II) are the most abundant and toxic principles (Fig. 1) (Kumar et al. 2003; Michl et al. 2014). Whereas both AA‐I and AA‐II induce aristolactam(AL)‐DNA adducts (Schmeiser et al. 1988; Pfau et al. 1990b; Shibutani et al. 2007), mutations (Schmeiser et al. 1990; Schmeiser et al. 1991; Xing et al. 2012) and carcinogenesis in rodents (Mengs et al. 1982; Mengs 1988), only AA‐I shows strong nephrotoxic effects in mice (Sato et al. 2004; Shibutani et al. 2007). In the literature, the mixture of AA‐I and AA‐II is often designated as AA.

In humans, AA ingestion is associated with aristolochic acid nephropathy (AAN) (Vanherweghem et al. 1993; Gillerot et al. 2001) and its environmental form known as Balkan endemic nephropathy (BEN) (Grollman et al. 2007). One of the unique features of AAN is its strong association with malignancies of the upper urinary tract (Cosyns et al. 1999; Nortier et al. 2000; Chen et al. 2012; Hoang et al. 2013). However, not all individuals are susceptible to the adverse effects of AA and there is a long latent period between exposure and effects (Vanherweghem 1998; Grollman 2013). Therefore, toxicities of AA in humans have long been overlooked. Recently, owing to the advances in mass spectrometry and next generation sequencing techniques, AA ingestion has been implicated in the etiology of renal cell carcinoma (Scelo et al. 2014; Jelakovic et al. 2015; Hoang et al. 2016; Turesky et al. 2016), bladder cancer (Poon et al. 2015), intrahepatic cholangiocarcinoma (Zou et al. 2014), and hepatocellular carcinoma (Poon et al. 2013; Totoki et al. 2014; Ng et al. 2017). These findings indicate that exposure to AA contributes significantly to kidney disease and cancer worldwide.

Since *Aristolochia* species are common in Chinese Traditional Medicine, it is estimated that in China alone there are 10–100 million people at risk of developing AA‐associated kidney disease and cancer (Hu et al. 2004; Grollman 2013). In Taiwan, where one‐third of the population has been exposed to AA (Hsieh et al. 2008), the link between AA exposure, upper urinary tract cancer (Chen et al. 2012), and hepatocellular carcinoma (Ng et al. 2017; Chen et al. 2018) is well documented. Despite abundant warnings (Administration 2001; World Health Organization 2008; National Toxicology Program 2011), AA‐containing remedies are still marketed online and are available for purchase in many countries around the world, including the United Kingdom (Gold and Slone 2003), the European Union (Maggini et al. 2018), the United States (Vaclavik et al. 2014), and Australia (Tay 2019).

Approximately 5–10% of exposed individuals are prone to AA‐related diseases (Vanherweghem 1998; Bamias and Boletis 2008). Polymorphisms in genes encoding xenobiotic‐metabolizing enzymes may contribute to interindividual variation in sensitivity to the effects of toxicants. However, no clear association between such a candidate gene and AA‐induced disease has been demonstrated. Therefore, identifying the full set of enzymes responsible for the metabolism of AA will provide indispensable clues that will define individual sensitivity to the toxic effects of AA.

Nitroreduction (NR) of AA is necessary for the formation of reactive intermediates (Fig. 1) that bind covalently to the exocyclic amino groups of dA‐ and dG‐forming AL‐DNA adducts (Pfau et al. 1990c). The dA‐AL adduct is highly persistent (Fernando et al. 1993; Grollman et al. 2007) and mutagenic (Attaluri et al. 2010) accounting for the AA‐mutational signature that is observed in human cancers related to AA‐exposure (Grollman et al. 2007; Chen et al. 2012; Hoang et al. 2013; Hollstein et al. 2013). Several mammalian enzymes, including NAD(P)H: quinone oxidoreductase (NQO1), xanthine oxidase, cytochromes P450:1A1/2 (CYP1A1 and CYP1A2), CYP:P450 oxidoreductase, and prostaglandin H‐synthase activate AA to form DNA‐binding species (Stiborova et al. 2009). AA activation requires the transfer of four electrons and occurs, presumably, through the formation of *N*‐hydroxyaristolactams, AL‐I‐NOH, and AL‐II‐NOH (Pfau et al. 1990a; Pfau et al. 1990c).

We recently found that *N*‐hydroxyaristolactams react only weakly with DNA (Sidorenko et al. 2014), findings consistent with the low efficiency of NR activation of AA *in vitro*. Commonly, esterification to *O*‐acetylated and *O*‐sulfonated derivatives stimulates covalent binding of the *N*‐hydroxylated metabolites of nitroarenes and arylamines to DNA (Bartsch 1981; Glatt 2000; Purohit and Basu 2000). Nevertheless, the involvement of cytosolic sulfotransferases (SULTs) and N‐acetyltransferases (NATs) in AA bioactivation remains a matter of debate. First, it was shown that expression of human SULT1A1 and SULT1B1 in bacterial and human cells increases the mutagenicity of AA‐I (Meinl et al. 2006). Later, conflicting evidence was presented demonstrating that bioactivation of AA by human NQO1 is not enhanced by the presence of several recombinant SULTs and NATs (Stiborova et al. 2011). Aiming to resolve this apparent discrepancy, Sidorenko et al. reported that *O*‐sulfonation of AL‐I‐NOH and AL‐II‐NOH leads to the formation of highly reactive *N*‐sulfonyloxyester anions of AL‐I‐NOSO_3_H and AL‐II‐NOSO_3_H, respectively (Sidorenko et al. 2014). Subsequently, human SULT1B1, SULT1A1, SULT1A2, and SULT1A3 were found to be involved in AA activation *in vitro* and in cultured mammalian cells (Hashimoto et al. 2016). Nevertheless, the controversy regarding the enzymes involved in the bioactivation of AA remains, because it was reported that following AA‐I exposure, transgenic mice carrying the functional human *SULT1A1‐SULT1A2* gene cluster accumulate comparable levels of AL‐DNA to that detected in the parental strain deficient in the murine sult1a1 and sult1a2 (Arlt et al. 2016). Therefore, it is important to assess fully the mechanisms and enzymes involved in the activation of *N*‐hydroxyaristolactams, which latter we are capable of synthesizing on demand (Attaluri et al. 2014).

The majority of studies addressing AA genotoxicity were mainly focused on AA‐I, or a mixture of AA‐I and AA‐II. Although AA‐I and AA‐II are known mutagens in *Salmonella typhimurium* (*S*. *typhimurium*) strains efficient in NR (Schmeiser et al. 1984), no evidence regarding the mutagenic effects of *N*‐hydroxyaristolactams in bacteria has been presented thus far. Moreover, despite the preliminary evidence that *N,O*‐acetyltransfer may be involved in AA activation, this mechanism has not been pursued in detail. Previously, one of us has established various *S*. *typhimurium umu* tester strains that overexpress bacterial *O*‐acetyltransferase, human *N*‐acetyltransferases (NATs) or human sulfotransferases (SULTs) (Oda et al. 1985, 1993, 1995, 1999, 2012). The utility of these strains in evaluating the toxicities of xenobiotics has been validated by studying various nitroarenes and aromatic amines. In the present work, we examine the role of bacterial and human enzymes in the genotoxicities of AL‐I‐NOH and AL‐II‐NOH using SOS‐based assay in *umu* tester strains. Furthermore, ^32^P‐postlabeling DNA adduct analysis (Reddy and Randerath 1986; Shibutani et al. 2006) is employed to study the bioactivation mechanisms of the *N‐*hydroxyaristolactams by means of cytosolic extracts obtained from cultured human kidney HK‐2 cell line (Ryan et al. 1994; Hashimoto et al. 2016).

## MATERIALS AND METHODS

### Chemicals and Enzymes

AL‐I‐OH and AL‐II‐NOH were synthesized in our laboratory as described (Attaluri et al. 2014), and AA‐I was purified from *A. indica* by high‐performance liquid chromatography. AA‐related compounds were dissolved in dimethyl sulfoxide (DMSO) at 30–40 mM and stored at −20°C. 2,6‐Dichloro‐4‐nitrophenol (DCNP) was purchased from Tokyo Chemical Industry Co. LTD (Japan). Enzymes used for ^32^P‐postlabeling analysis were obtained from Worthington (Newark, NJ), New England Biolabs (Ipswich, MA), MP Biomedicals (Solon, OH), and Sigma Aldrich (St. Louis, MO). DMSO, 3′‐phosphoadenosine‐5′‐phosphosulfate (PAPS) (70% purity), salmon sperm DNA (ssDNA), pentachlorophenol (PCP), quercetin, β‐estradiol, 4‐nitrophenol were from Sigma‐Aldrich. PAPS (>90% purity) and 3′‐phosphoadenosine‐5′‐phosphate (PAP) were purchased from R&D Systems (Minneapolis, MN). dG‐AL‐II and dA‐AL‐II containing oligonucleotides were synthesized as described (Attaluri et al. 2010). γ‐^32^P‐ATP (6000 μCi/mmol) was obtained from PerkinElmer (Boston, MA). Recombinant human SULT1B1 was purchased from MyBioSource Inc. (San Diego, CA).

### Bacterial Strains

The following *S. typhimurium umu* tester trains were used: NM2009 (bacterial O‐acetyltransferase (*O*‐AT)‐overproducing strain), TA1535/pSK1002 (parental strain), and NM2000 (*O*‐AT‐deficient strain) (Oda et al. 1985, 1993); NM6000 (*O*‐AT‐deficient parental strain), NM6001 (human NAT1 expressing *strain*), NM6002 (human NAT2‐expressing strain) (Oda et al. 1999); and NM7000 (*O*‐AT‐deficient parental strain), NM7001 (human SULT1A1‐expressing strain), NM7002 (human SULT1A2‐expressing strain), and NM7003 (human SULT1A3‐expressing strain) (Oda et al. 2012).

### Genotoxicity (SOS/*umu)* and Cytotoxicity Assays Involving TA1535/pSK1002, NM2009, NM2000, NM6000, NM6001 and NM6002 Strains

*Umu* assay was carried out as described (Oda et al. 1995). Briefly, after overnight culture, each strain was diluted 100‐fold with TGA medium containing 1% bactotryptone (w/v), 0.5% NaCl (w/v), 0.2% glucose (w/v), 20 μg/mL ampicillin, and incubated on a shaker for 1 h at 37°C. After cell density reached an absorbance of 0.25–0.3 at 600 nm, 10 μL of AL‐I‐NOH, or AL‐II‐NOH dissolved in DMSO was added. The resulting mixtures were incubated for 2 h as above. Induction of *umuC* gene expression as a response to DNA damage was determined by measuring cellular β‐galactosidase activity as reported previously (Oda et al. 1985). Values obtained for β‐galactosidase activity for each dose of *N*‐hydroxyaristolams were divided by that recorded in the absence of the compounds and compared to the fold‐changes observed using respective parental strains. The cytotoxic effects of the *N*‐hydroxyaristolactams were determined by monitoring the changes in the optical density of the cells at 600 nm. Genotoxicity and cytotoxicity results are presented as mean values of three independent experiments.

### *Umu* Assay in Strains Expressing Human SULTs

The *umu* assay was conducted as described previously (Oda et al. 2012). Briefly, bacterial cells were grown overnight at 37°C in LB broth containing ampicillin (25 μg/mL) and kanamycin (25 μg/mL). The cultures were diluted 50‐fold with TGA medium, supplemented with 1 mM isopropyl‐α‐D‐thiogalactoside and incubated at 37°C for 3 h until the cell density reached an absorbance of ~0.3 at 600 nm. Aliquots (1 mL) of the TGA culture and 10 μL of compounds dissolved in DMSO were mixed and further incubated with agitation for 3 h at 37°C. Genotoxicities and cytotoxicities of compounds were evaluated and presented as described above.

### HK‐2 Cell Culture and Preparation of Cytosolic Fractions

A human kidney HK‐2 cell line was purchased from the American Type Culture Collection (ATCC, Manassas, VA) and cultured in K‐SFM media as recommended by the manufacturer under 5% CO_2_ at 37°C. ATCC validates cell origin by Short Tandem Repeat analysis and conducts testing of cells for mycoplasma contamination prior to release of the product to customers.

Cells from confluent cultures grown on 75 cm^2^ plates were collected, and cytosolic lysates were prepared by homogenization and ultracentrifugation in Tris–HCl pH 7.5 supplemented with inhibitors of proteases (Roche, Branchburg, NJ), as described for renal and hepatic murine tissues (Sidorenko et al. 2014). Alternatively, cellular fractions were prepared by using the nuclear cytosolic fractionation kit following manufacturer's instructions (Cell Biolabs, San Diego, CA). Half of each cytosolic preparation was divided in small aliquots and stored at −80°C until used in an activation assay. The rest of the sample was dialyzed against Tris–HCl (pH 7.5) on membranes with 10 kDa or 3.5 kDa molecular weight cut off (Thermo Scientific, Rockford, IL). Two dialysis schemes for each molecular weight cut off were conducted. Depending on the size of the membrane, sample volume was 100 μL, for Slide‐A‐Lyzer MINI Dialysis Unit, or 1 mL, when using Slide‐A‐Lyzer Dialysis Cassette. The dialysis buffer was used at 500 times the volume of the sample. A typical procedure was as follows: dialysis for 2 h at 4°C followed by change of the buffer and dialysis for additional 2 h. Following these two steps, the buffer was replaced with the fresh portion and samples were further dialyzed overnight at 4°C. Dialysis experiments were reproduced several times on different days, which did not affect the outcomes and conclusions of this study. Protein content before and after dialysis was measured by the bicinchoninic acid assay or/and by the Bradford assays. Results obtained by both assays were consistent. Dialyzed samples were handled and stored the same way as nondialyzed.

### Activation of *N*‐Hydroxyaristolactams by HK‐2 Cytosols

Unless indicated otherwise, the following components were included in each 100 μL of incubation mixture: 80 μg of ssDNA in 50 mM Tris–HCl pH 7.5, 0.5 mM EDTA, 15 mM MgCl_2_, and 1–100 μM AL‐I‐NOH. To study the effects of cofactors, either of the following compounds was added to mixtures at the following concentrations: 0.2 mM PAPS, 1 mM acetyl‐CoA, 1 mM NADPH, 1 mM NADH, 1 mM FAD, and 1 mM FMN (all from Sigma‐Aldrich). When necessary, various compounds (pentachlorophenol, 4‐nitrophenol, PAP, quercetin, β‐esrtadiol) were included in the reaction mixtures in the dose range of 0.1–1000 μM. Reactions were initiated by the addition of cytosols and incubated at 37°C for 15 min‑4 h. Generally, we conducted serial dilutions of cytosolic protein to obtain a dose range between 6 and 25,000 ng/100 μL. Details are provided in the corresponding figures. At the indicated time, 100 μL volume was withdrawn from each reaction mixture and immediately combined with the equal volume of phenol‐isoamyl‐alcohol followed by rigorous vortexing for 15 s. Proteins were extracted by three steps of repeated shaking with phenol‐isoamyl‐alcohol, followed by centrifugation. DNA from aqueous upper fractions was precipitated by a standard procedure using ice‐cold ethanol and sodium acetate at acidic pH. DNA pellets were dissolved in water and the yields were quantified by measuring the optical density of the samples at 260 nm. Collected DNA samples were stored at −20°C until adduct analysis as below. Control incubations were run routinely and were as follows: (1) without ssDNA, (2) without AL‐NOH, (3) without cofactors, or (4) without cytosols. Each reaction was conducted in triplicate and/or repeated on a different day with new aliquots of cytosols and new dilutions of AL‐I‐NOH.

### ^32^P‐Postlabeling Adduct Analysis

DNA adduct levels were evaluated as described previously (Dong et al. 2006; Sidorenko et al. 2012; Hashimoto et al. 2016). Briefly, DNA samples (5 or 10 μg) were enzymatically digested to 3′‐phosphate nucleosides. The resulting AL‐adducted nucleosides were enriched by butanol extraction, and the mixtures were evaporated to dryness by centrifugation under vacuum. Samples were reconstituted with water and labeled by radioactive phosphorus, transferred from γ‐^32^P‐ATP to 5′‐hydroxyl groups of nucleosides by polynucleotide kinase deficient for 3′‐phosphatase activity. Following labeling, the samples were dried as above and reconstituted in the loading buffer containing formamide and bromphenol blue. The labeled products were resolved by gel electrophoresis using 30% nondenaturing polyacrylamide (PAGE). The following synthetically obtained oligonucleotides (Attaluri et al. 2010; Sidorenko et al. 2012) were digested, as a mixture of 15, 30, or 60 fmol of each, and processed in parallel with DNA samples obtained from activation assay:

5’‐TCT TCT TCT GTG CXC TCT TCT TCT‐3’ X = dA‐AL‐II

5’‐TCT TCT TCT GTX CAC TCT TCT TCT‐3’ X = dG‐AL‐II

Gels were exposed to a phosphor screen (GE Healthcare) for various times (5 min—several hours, depending on adduct levels). The results were visualized by the Typhoon system and densitometry was conducted using Image QuaNT v5.2 (Molecular Dynamics). Representative fragments of PAGE are presented. AL‐DNA adduct levels were plotted in Sigma Plot v13.0 (SPSS Inc.) and shown as mean values and standard deviations obtained from triplicate experiments, or as values obtained from the most representative experiment.

## RESULTS

### Cytotoxicity and Genotoxicity of AL‐I‐NOH and AL‐II‐NOH in TA1535/pSK1002, NM2009, and NM2000 *S. typhimurium* Strains

**Figure 2:**
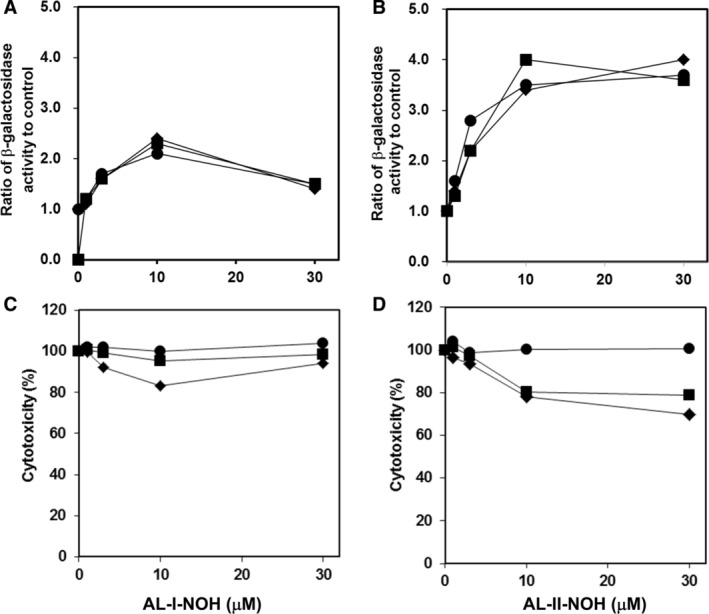
Induction of *umuC* gene expression (A and B) and cytotoxicity response (C and D) induced by AL‐I‐NOH and AL‐II‐NOH in *S. typhimurium* tester strains TA1535/pSK1002 (◆), NM2009 (■) and NM2000 (●). Experiments were carried out as described in the section Material and Methods. In (A) and (B), each point was derived as the ratio of β‐galactosidase activity for each dose of the compound to the activity in the corresponding untreated cells. In all graphs, points represent mean values of triplicate determinations.

To assess whether bacterial *O*‐acetyltransferases (*O*‐AT) are involved in the genotoxicities of the *N*‐hydroxyaristolactams, we employed NM2009 (bacterial *O*‐AT overexpressing strain), NM2000 (*O*‐AT deficient strain) and TA1535/pSK1002 (parental strain) *umu* tester strains. Background *β*‐galactosidase activity in the absence of compounds was evaluated for each strain and indicated as “1” on the *y*‐coordinate (Fig. 2A, B). For each tester strain, any multifold changes in the baseline activity of β‐galactosidase in response to treatments with *N*‐hydroxyaristolactams are presented as functions of compound concentrations. The SOS/*umu* test showed that both *N*‐hydroxyaristolactams are genotoxic in all three strains, with AL‐II‐NOH being more genotoxic than AL‐I‐NOH (Fig. 2A, B). Importantly, changes in β‐galactosidase activities in NM2009 and NM2000 cells were similar to those found in the parent strain. In addition, AL‐II‐NOH was slightly cytotoxic (~20%–30% reduction in cell growth) to TA1535/pSK1002 and NM2009 cells but did not affect NM2000 strain (Fig. 2D), whereas AL‐I‐NOH was mildly cytotoxic only to the parental strain (Fig. 2C). These results suggest that bacterial *O*‐acetyltransferases are not important for the genotoxicities of the *N*‐hydroxyaristolactams.

### Effects of AL‐I‐N‐OH and AL‐II‐NOH on Cytotoxicity and *umuC* Induction in the *S. typhimurium* Tester Strains: NM6000, NM6001, NM6002, NM7000, NM7001, NM7002, and NM7003

**Figure 3:**
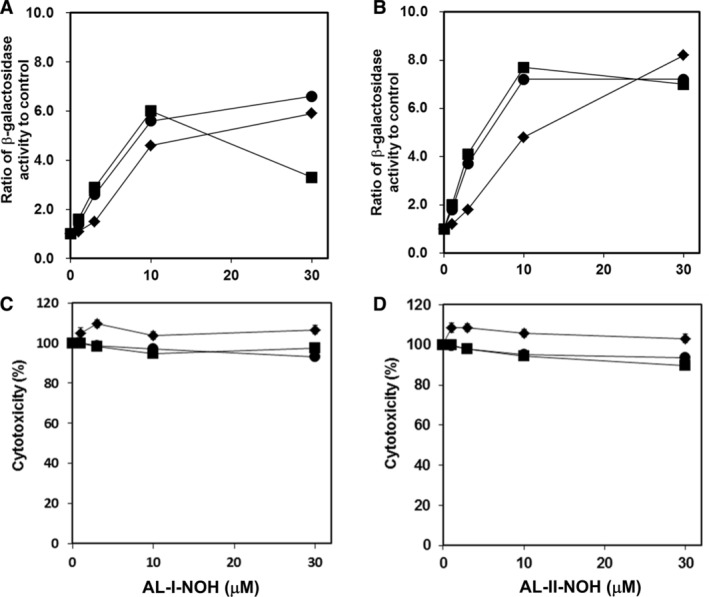
Induction of *umuC* gene expression (A and B) and cytotoxicity response (C and D) induced by AL‐I‐NOH and AL‐II‐NOH in *S. typhimurium* tester strains NM6000 (◆), NM6001 (■), and NM6002 (●). In (A) and (B), results are presented as values corresponding to β‐galactosidase activity normalized to untreated control. In all graphs, points are mean values of triplicate determinations.

NM6001 (human NAT1 expressing), NM6002 (human NAT2 expressing), and NM6000 (parental) *umu* strains were used to examine the involvement of human NATs in toxicities of the *N*‐hydroxyaristolactams. Background *β*‐galactosidase activity in all strains, established in the absence of *N*‐hydroxyaristolactams, increased with increasing concentration of either compound, AL‐I‐NOH and AL‐II‐NOH (Fig. 3A, B). Although, the parental strain, lacking human enzymes, also responded to exposure, the effects were more pronounced in NAT‐producing strains. Thus, in both NAT‐expressing strains, the presence of 3 μM AL‐I‐NOH or AL‐II‐NOH increased induction of the *umuC* gene by 2.6‐ or 4.1‐fold, respectively. In the NM6000 strain, the same concentration of compounds induced 1.5‐ (AL‐I‐NOH) and 1.8‐fold (AL‐II‐NOH) changes relative to their corresponding background β‐galactosidase activities (Fig. 3A, B). We also found that AL‐II‐NOH was more genotoxic than AL‐I‐NOH across all three strains. With respect to cytotoxicity, AL‐I‐NOH at the dose range up to 30 μM did not induce significant toxicity in these *umu* strains (Fig. 3C), whereas AL‐II‐NOH was slightly cytotoxic showing approximately a 10% reduction in cell number in NAT1‐ and NAT2‐expressing strains (Fig. 3D). These results indicate that expression of human NAT1 and NAT2 renders bacterial cells sensitive to the genotoxic effects of AL‐I‐NOH and AL‐II‐NOH.

**Figure 4:**
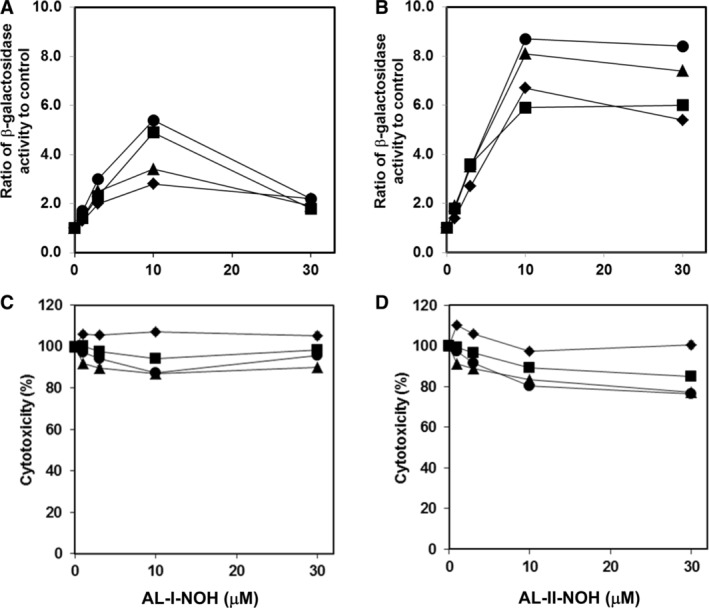
Induction of *umuC* gene expression (A and B) and cytotoxicity response (C and D) induced by AL‐I‐NOH and AL‐II‐NOH in *S. typhimurium* tester strains NM7000 (◆), NM7001 (■), NM7002 (●), and NM7003 (▲). Experiments were conducted as described in the section Material and Methods. In (A) and (B), values for β‐galactosidase activity, observed for each dose of compound, were divided by that measured in corresponding untreated strain. In all graphs, points represent mean values of triplicate determinations.

Subsequently, to study the role of human SULTs in the bioactivation of AL‐I‐NOH and AL‐II‐NOH, we evaluated *umuC* gene expression and cytotoxicity in response to these compounds in the following four tester strains: parental (NM7000) strain and strains expressing human SULT1A1 (NM7001), SULT1A2 (NM7002), and SULT1A3 (NM7003) isoforms. The background and *N*‐hydroxyaristolactams‐induced activities of β‐galactosidase in all four strains were established and presented (Fig. 4A, B) as described above for other bacterial strains. In the presence of 10 μM AL‐I‐NOH, the background activities of *umuC* gene were increased, respectively, by 4.9‐ and 5.4‐fold in SULT1A1 and SULT1A2‐expressing strains (Fig. 4A). Under the same conditions, AL‐I‐NOH led to 2.5‐ and 2.8‐fold elevated activities of β‐galactosidase in the parental and SULT1A3‐producing cells, respectively, indicating that SULT1A3 may not be involved in the genotoxicity of AL‐I‐NOH. Overall, AL‐II‐NOH was more genotoxic than AL‐I‐NOH across all four tester strains. Thus, this compound at 10 μM induced an approximate 8‐fold increase in β‐galactosidase activity in both SULT1A2‐ and SULT1A3‐expressing strains. The amplitude of response in the parental and SULT1A1 strains showed about a 6‐fold difference from corresponding background values in untreated cells. With respect to cytotoxicity, both compounds were slightly cytotoxic in SULT1A‐expressing bacteria, showing maximally a 20% reduction in cell density compared to the value for untreated cells, but not that of the parental strain (Fig. 4C, D).

To provide further evidence for the importance of SULTs in the genotoxicities of *N*‐hydroxyaristolactams in SULT‐expressing bacteria, we evaluated the effects of 2,6‐dichloro‐4‐nitrophenol (DNCP), an inhibitor of SULT1A (Seah and Wong 1994), on the *umuC* gene expression induced by AL‐I‐NOH and AL‐II‐NOH in the SULT1A2‐expressing strain (Table 1). The presence of 50 μM DNCP reduced genotoxicities of AL‐I‐NOH and AL‐II‐NOH by 2.3‐ and 2.1‐fold, respectively. These results corroborate our previous observations that human SULT1A enzymes are involved in the bioactivation of *N*‐hydroxyaristolactams. Intriguingly, in all of our parental strains, we detected the activity of β‐galactosidase, which showed dose dependence in response to the treatment with *N*‐hydroxyaristolactams, thus suggesting that they induce other mechanisms of mutagenesis in bacterial and, potentially, mammalian cells.

**Table 1:** The Effect of 2,6‐Dinitro‐4‐Nitrophenol on The Induction of *UmuC* Gene Expression by AL‐I‐NOH and AL‐II‐NOH in *S. typhimurium* NM7002

Inhibitor	Activation of chemicals, β‐Galactosidase activity (units) (%)	
	AL‐I‐NOH	AL‐II‐NOH
None	355 ± 13 (100)	621 ± 14 (100)
DNCP^a^	156 ± 6 (44)	292 ± 32 (47)

a DNCP: 2,6‐dinitro‐4‐nitrophenol.

Incubations of cells were conducted with or without 50 μM DCNP in the presence of AL‐I‐NOH (10 μM) or AL‐II‐NOH (20 μM). The induction of *umuC* gene expression was determined as described in the section Materials and Methods. Experiments were carried out in triplicate for each condition, and the results are presented as mean values ± standard deviation. Percent of β‐galactosidase activity from control incubations without DCNP is shown in parenthesis.

### Bioactivation of *N*‐Hydroxyaristolactams in AL‐DNA by HK‐2 Cytosols

In order to explore mechanisms of bioactivation of *N*‐hydroxyaristolactams in human cells, we studied their activation in AL‐DNA in the presence of ssDNA and cytosolic extracts obtained from cultured immortalized human kidney HK‐2 cells. The basis for the bioactivation assay of *N*‐hydroxyaristolactams was previously established by one of us (Sidorenko et al. 2014) for murine renal and hepatic cytosols. In this type of experiment, proteins and cofactors present in cell lysates are capable of transforming low reactive *N*‐hydroxyaristolactams into highly reactive species, which bind DNA covalently forming AL‐DNA adducts according to the mechanism outlined in the Figure 1.

**Figure 5:**
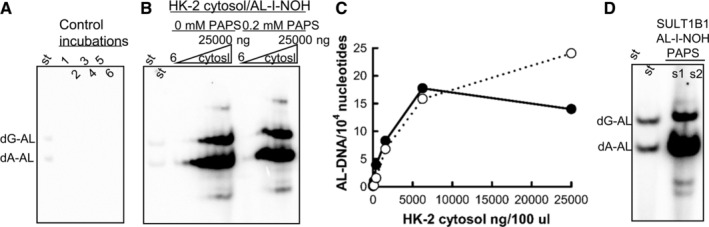
Activation of AL‐I‐NOH in AL‐DNA by HK‐2 cytosols, fortified, and not fortified by PAPS. Cytosols from HK‐2 cells (6; 25; 100; 400; 1500; 6250 and 25,000 ng/100 μL) or recombinant SULT1B1 protein (1 ng/100 μL) were incubated with 100 μM AL‐I‐NOH in the presence of ssDNA with or without 0.2 mM PAPS for 2 h at 37°C. DNA was extracted and two micrograms from each sample was subjected to adduct analysis as described in the section Materials and Methods. (A), (B), and (D) are representative fragments of polyacrylamide gels following electrophoresis and exposure for 2 (A and B) or 10 min (D) on a phosphoscreen. (A) and (B) are unaltered fragments of the same gel shown at the same contrast, and analysis in (D) was done in a separate experiment and shown at the similar contrast level but longer exposure time. Control 1 in (A) is DNA/cytosol; Control 2—DNA/AA‐I; Controls 3–5—DNA/AL‐I‐NOH; Control 6—DNA/AL‐I‐NOH/PAPS. AL‐DNA levels in control incubations with AL‐I‐NOH were at 0.2 ± 0.02 adducts/10^6^ nucleotides. (C) Quantitative representation of results shown in (B). Filled circles—reactions with PAPS; empty circles—without PAPS. (D) s1 (8 AL‐DNA/10^5^ nucleotides) and s2 (6 AL‐DNA/10^5^ nucleotides) represent adducted DNA from reactions conducted with PAPS from R&D and Sigma‐Aldrich, respectively. St—standard mixture of oligonucleotides containing dG‐AL‐II and dA‐AL‐II adducts, 30 fmol each. AL‐DNA is a combined term for dG‐AL and dA‐AL adducts.

**Figure 6:**
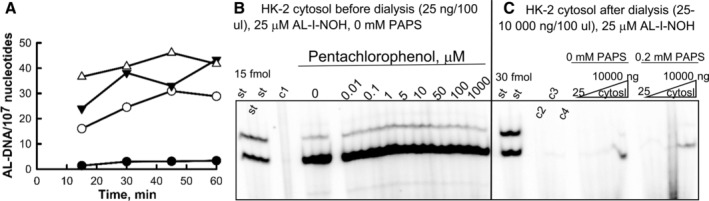
Effect of dialysis on 10 kDa MWCO and pentachlorophenol on activation of AL‐I‐NOH in AL‐DNA by HK‐2 cytosols. (A) Cytosols from HK‐2 cells (25 ng/100 μL) were incubated with 1 (filled circles), 10 (empty circles), 25 (filled triangles), or 50 μM (empty triangles) AL‐I‐NOH in the presence of ssDNA for 15–60 min at 37°C. DNA was extracted and five micrograms from each sample was subjected to adduct analysis as described in the section Materials and Methods. Results are presented as the dependence of AL‐DNA (dG‐AL and dA‐AL adducts) levels on the reaction time. (B) HK‐2 cytosols were incubated with AL‐I‐NOH and ssDNA in the presence and absence of pentachlorophenol (PCP) for 30 min as indicated. Each reaction with PCP was conducted in duplicate, and reaction without PCP was set in triplicate. A fragment of representative PAGE with DNA adduct analysis for five microgram of DNA is shown. AL‐DNA levels without PCP were 58 ± 4 adducts per 10^7^ nucleotides, and 60 ± 4 with PCP, combined across all concentrations of PCP. c1‐ssDNA incubated with AL‐I‐NOH. C. HK‐2 cytosol was dialyzed on 10 MWCO membrane against Tris–HCl buffer (pH 7.5) overnight at 4°C. The protein amount was quantified by Bradford assay, and various amount of protein (25 ng, 50 ng, 200 ng, 1000, 5000, 10,000 and 25,000/100 μL) was incubated with ssDNA and AL‐I‐NOH with or without PAPS for 30 min. c2—DNA; c3—DNA, AL‐I‐NOH; c4—DNA, AL‐I‐NOH, PAPS. St‐ standard mixture of oligonucleotides containing dG‐AL‐II and dA‐AL‐II adducts, 15 and 30 fmol each. AL‐DNA levels were 0.3 (25 ng protein) and 8 (10,000 ng protein) adducts per 10^7^ nucleotides, which is ~500‐times less than before dialysis. Reactions in (B) and (C) were conducted in parallel and analyzed in the same digestion assay and resolved on the same gel. The vertical line was introduced manually to indicate the border between two different experiments. Upper band is dG‐AL, lower band is dA‐AL.

Consistent with our previous reports, AL‐I‐NOH by itself showed only weak reactivity toward DNA (Fig. 5A). HK‐2 cytosols prepared by ultracentrifugation efficiently activated AL‐I‐NOH in dG‐AL‐I and dA‐AL‐I in the presence of ssDNA. This activity showed linear dependence on the protein amount (Fig. 5C) and the concentration of AL‐I‐NOH (Fig. 6A). In the presence of 6 ng of cytosolic proteins per 100 μL of reaction mixture, 10 AL‐DNA adducts per 10^6^ nucleotides were formed in 2 h. This number was raised by 500 times in the presence of 25 μg of the protein, resulting in 15‐20 AL‐DNA per 10^4^ nucleotides, suggesting that at least 2% of AL‐I‐NOH was transformed in AL‐DNA (Fig. 5C). Surprisingly, addition of PAPS, cofactor of SULTs, did not stimulate this activity (Fig. 5B, C). Because similar results were found for AL‐II‐NOH (not shown), we used AL‐I‐NOH in all subsequent experiments. To ensure that PAPS, cofactor for SULTs, was active, its quality was evaluated in the activation assay of AL‐I‐NOH by SULT1B1 (Fig. 5D), showing that the presence of PAPS efficiently induces AL‐DNA when AL‐I‐NOH was incubated with SULT1B1 and DNA. PAPS obtained from Sigma‐Aldrich or R&D systems having different purities, >90% and > 70%, respectively; induced similar adduct levels, namely six and eight AL‐DNA adducts per 10^5^ nucleotides, respectively.

**Figure 7:**
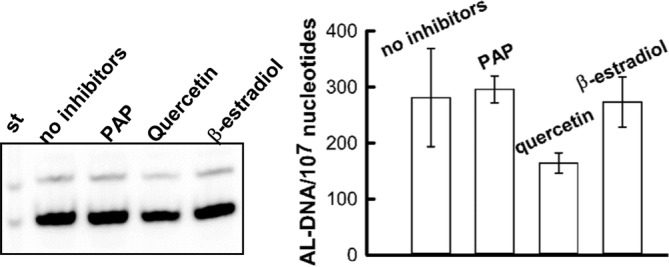
Effect of PAP, quercetin, and b‐estradiol on activation of AL‐I‐NOH in AL‐DNA by HK‐2 cytosols. Cytosols from HK‐2 cells (25 ng/100 μL) were incubated with 50 μM of AL‐I‐NOH in the presence of ssDNA for 30 min at 37°C. Reactions were set in triplicate for each condition, without or with 100 μM one of the following: PAP, quercetin and β‐estradiol. DNA was extracted and five micrograms from each sample was subjected to adduct analysis. Fragment of PAGE and quantification of obtained results are shown. St‐ mixture of standard at 30 fmol each. dG‐ and dA‐AL are upper and lower bands, respectively.

To ensure the linearity of adduct formation for further experiments with various inhibitors and potential cofactors of bioactivation, we evaluated the effect of the protein amount, AL‐I‐NOH dose and incubation time on the rates of AL‐DNA formation. The reaction followed a linear time course up to 30–45 min (Fig. 6A). With respect to the protein and AL‐I‐NOH amounts, 25 ng protein per 100 μL reaction mixture and 25–50 μM AL‐I‐NOH produced DNA adducts at the levels of 20–40 adducts per 10^7^ nucleotides (Fig. 6A). Under established conditions, there was no apparent inhibition of HK‐2 mediated bioactivation of AL‐I‐NOH in the presence of pentachlorophenol (PCP, Fig. 6B) or 4‐nitrophenol (not shown). Both compounds were evaluated in a concentration range of 0.01–10,000 μM, no inhibition being observed. Also, neither PAP nor β‐estradiol affected the levels of AL‐DNA (Fig. 7). However, quercetin, an inhibitor of SULT1A family, at 100 μM inhibited AL‐DNA levels by two fold (Fig. 7).

**Figure 8:**
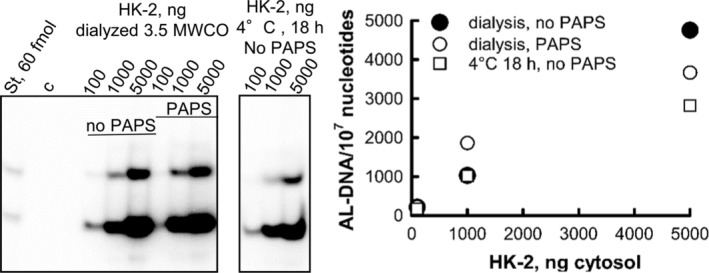
Effect of dialysis on 3.5 kDa MWCO and prolonged incubation at 4°C on activation of AL‐I‐NOH in AL‐DNA by HK‐2 cytosols. Cytosols from HK‐2 cells were dialyzed against Tris–HCl buffer (pH 7.5) using 3.5 MWCO membrane overnight at 4°C. In parallel, a portion of cytosol was incubated above the membrane without the dialysis overnight at 4°C. 100–5000 ng of the protein was incubated with ssDNA and 100 μM AL‐I‐NOH with or without PAPS, as indicated. Reactions were allowed to run for 45 min at 37°C. DNA (5 μg) was used for adduct analysis. Fragments of PAGE (left panel) and quantification of obtained results (right panel) are shown. St‐ mixture of standard at 60 fmol each. Upper band is dG‐AL, and lower band is dA‐AL.

Subsequently, we dialyzed HK‐2 cytosols using membranes with a 10 kDa molecular weight cutoff and found that following dialysis the activity of cytosols dropped drastically, showing a 500‐fold reduction as compared to the HK‐2 sample before dialysis. This activity could not be restored by the addition of PAPS (Fig. 6C). Moreover, neither of the other compounds, alone or introduced as a mixture, including AcCoA, NADH, NADPH, FAM, or FNM was capable of restoring AL‐I‐NOH activation (data not shown). In contrast, dialysis on 3.5 kDa units reduced this activity only by half and addition of PAPS had no effect on the reaction rate (Fig. 8). To assess whether prolonged incubation at 4°C affects the AL‐DNA formation, we incubated the cytosolic fractions on the dialysis membrane overnight at 4°C and found no difference in activity between this fraction and the dialyzed sample, indicating that reduction in the bioactivation activity of cytosolos following incubation on 3.5 kDa units results from defrosting and/or subsequent prolonged incubation of the protein sample in cold. These results suggest that there is an unidentified mechanism(s) capable of promoting AL‐DNA formation mediated by AL‐I‐NOH and HK‐2 cytosols.

## DISCUSSION

### Summary of Results

*N*‐hydroxyaristolactams are formed by a four‐electron reduction process of the nitro group of the AA followed by intramolecular modifications (Priestap et al. 2010; Priestap et al. 2012). In this article, we investigate mechanisms underlying the genotoxicities of the *N*‐hydroxyaristolactams in bacterial and human kidney cells. First, we evaluated the genotoxicities of AL‐I‐NOH and AL‐II‐NOH using *umu* assay in *S. typhimurium* tester strains, which express bacterial *O*‐AT, or various isoforms of human NATs and SULTs. Furthermore, we employed cytosolic fractions obtained from human HK‐2 cells to separate the pathways of bioactivation of AL‐I‐NOH in mammalian cells. Based on our results, we conclude the following: (1) bacterial *O*‐AT are not involved in genotoxicity of AL‐I‐NOH and AL‐II‐NOH in *Salmonella* cells, (2) human NAT1, NAT2, SULT1A1, and SULT1A2 increase genotoxicities of AL‐I‐NOH and AL‐II‐NOH in *S. typhimurium*, whereas (3) human SULT1A3 is important for activation of AL‐II‐NOH but does not play a major role with respect to AL‐I‐NOH genotoxicity, and (4) additional mechanisms are implicated in the genotoxicities of these compounds in bacterial and mammalian cells.

### NR and Genotoxicities of AA

The toxicities of the AA are closely related to the stepwise reduction of their nitro group. NR may follow different routes and result in various outcomes depending on the nature of NR enzymes and oxygenation of tissues responsible for biotransformation (Purohit and Basu 2000). Thus, four‐electron reduction of AA eventually results in electrophilic cyclic nitrenium aristolactam ions (Pfau et al. 1990a; Pfau et al. 1990c) that bind DNA covalently yielding highly mutagenic and persistent dA‐AL adducts (Fernando et al. 1993; Grollman et al. 2007; Attaluri et al. 2010; Schmeiser et al. 2014). In the classical Ames assay, AA‐I is mutagenic in *Salmonella* strains that are efficient in NR (Schmeiser et al. 1984), whereas the aristolactam—six electron reduction products of AA—are genotoxic in these strains only in the presence of hepatic S9 microsomal fractions (Schmeiser et al. 1986). Therefore, based on these and other observations (Pfau et al. 1990c), it was suggested that reductive and oxidative metabolism of AA and aristolactams, respectively, result in *N*‐hydroxyaristolactams, which spontaneously decompose to cyclic nitrenium ions, yielding covalent adducts with DNA, and resulting in mutagenesis in bacterial and mammalian cells. In contrast to this hypothesis, we recently demonstrated that *N*‐hydroxyaristolactams *in vitro* react only weakly with DNA and require further activation by human SULTs (Sidorenko et al. 2014; Hashimoto et al. 2016). *However, these compounds display dose‐dependent genotoxic responses in our umu strains that lack bacterial O‐acetyltransferases and other parental strains that do not express human conjugation enzymes*. The single‐electron reduction mechanism and the existence of additional activation mechanism(s) involving conjugation of the *N‐O*‐group, but not carried by SULTs and NATs, may account for this discrepancy. These possibilities are discussed below.

### The Role of Single‐Electron Reduction and Oxidative Stress in AA Effects

One‐electron step‐wise reduction of various nitroaromatic compounds produces a nitro anion radical, known to engage in the redox cycle with molecular oxygen with concomitant formation of hydrogen peroxide, resulting in depletion of cellular nucleophiles and oxidative DNA damage (Purohit and Basu 2000; Boelsterli et al. 2006). Exposure to *N*‐hydroxylamines has also been implicated in oxidative stress and mutagenesis in bacterial and mammalian cells (Purohit and Basu 2000; Boelsterli et al. 2006). Although reduction of the AA by mammalian Type I two‐electron reductases, such as NQO1, is not oxygen sensitive, bacterial nitroreductases, and several other mammalian enzymes, known to activate AA, operate by a single‐electron mechanism (Stiborova et al. 2013; Hashimoto et al. 2016). However, the evidence for the involvement of oxidative stress in the toxicities of AA in mammalian cells is limited (Yu et al. 2011; Romanov et al. 2015; Wu et al. 2015), and the AA‐mutational signature in human tumors is strongly associated with dA‐AL adducts lacking the signatures known for oxidative DNA damage (Hoang et al. 2013; Alexandrov et al. 2015; Hoang et al. 2016; Petljak and Alexandrov 2016; Rosenquist and Grollman 2016). Accordingly, it appears that in mammalian cells formation of AL‐DNA through the cyclic nitrenium ion is the key step for AA‐genotoxicity and mutagenesis, whereas in facultative anaerobes, such as *S. thyphimurium* in the active growth stage, both oxidative DNA damage and AL‐DNA may contribute to the genotoxicities of AA and *N*‐hydroxyaristolactams. In contrast to most common *Salmonella* Ames strains, which are sensitive to either deletions, intercalations, or base substitutions at G46, our *umu* strains easily detect a wide range of genotoxic events that induce expression of the *umu* operon (Oda et al. 1985). *Therefore, in our experiments, genotoxicities of N‐hydroxyaristolactams in S. typhimurium result not only from the AL‐DNA formation and also from oxidative stress*. To fully assess the role of oxidative pathways in the genotoxicities of the *N*‐hydroxyaristolactams in bacterial cells, it will be important to study their mutagenicity in anaerobically grown bacteria, and bacteria lacking either classical nitroreductases or an 8‐oxoguanine DNA repair system.

### Involvement of Conjugation Reactions in Genotoxicities of AA

Until recently, it was believed that *N*‐hydroxyaristolactams, due to their instability were the major source of the cyclic nitrenium ions (Schmeiser et al. 1986; Pfau et al. 1990c;b). Indeed, some reductively activated nitroarenes react with cellular nucleophiles without further activation (Purohit and Basu 2000; Boelsterli et al. 2006). However, this reactivity can be amplified by *N‐O*‐conjugation. The most common esterification reactions of the hydroxylamines are carried by bacterial *O*‐acetyltransferases, human NAT1 and NAT2, and various human SULT enzymes yielding *N*‐*O*‐acetyl and *N*‐O‐sulfonyl esters (Hein et al. 1993; Oda et al. 1993; Oda et al. 1999; Glatt 2000; Oda et al. 2012). These transformations are enzyme‐, xenobiotic‐, and species‐specific and lead to the formation of easily excreted and, in many cases, highly unstable and reactive metabolites. Consistently, Meinl et al. (2006) reported that expression of human SULT1A1 in *Salmonella* and human cells moderately enhances the mutagenicity of AA‐I. In contrast, follow‐up studies using recombinant enzymes in conjunction with adduct analysis suggested that human NATs and SULT1A enzymes do not play a major role in AA‐I bioactivation, if initiated by cytosolic renal or hepatic extracts or recombinant NQO1 (Stiborova et al. 2011). To resolve this controversy, our collaborators synthesized *N*‐hydroxyaristolactams, their sulfonated and acetylated derivatives, and we provided evidence that sulfonation likely is more important for AA‐I bioactivation than *N*,O‐acylation (Sidorenko et al. 2014). In this study, we found that ‐*O*‐acetyltransport specific to human NAT1 and NAT2, but not bacterial O‐ATs, may be an overlooked bioactivation mechanism of *N*‐hydroxyaristolactams. Thus, *S. thyphimurium* overexpressing bacterial *O*‐AT respond to *N*‐hydroxyaristolactams with a similar to the parental strain amplitude. In contrast, genotoxin‐induced expression of *umuC* gene is greatly stimulated in bacteria expressing human NAT1 and NAT2. Accordingly, in our first report regarding the role of conjugation reactions in bioactivation of *N*‐hydroxyaristolactams, we found that *N*‐acetoxyaristolactams bind DNA covalently displaying rates of adduct formation similar to those for *N*‐sulfonyloxyaristolactams (Sidorenko et al. 2014). However, only NAT2, but not NAT1, obtained from insect cells overexpressing human proteins, stimulated DNA adduct formation triggered by the presence of *N*‐hydroxyaristolactams, DNA and AcCoA. The levels of AL‐DNA in these reactions were two orders of magnitude lower than that observed for recombinant SULT1A1/2 and SULT1B1. Therefore, at that time, we concluded that sulfonation is the primary bioactivation route of AL‐I‐NOH and AL‐II‐NOH. However, the source of the enzymes in the *in vitro* studies was commercial and NAT enzymes were present as a complex mixture of proteins, and no positive control with other xenobiotics was conducted. Therefore, we may have previously underestimated the role of NATs in bioactivation of AA, and the current studies provide evidence that *N,O*‐acetyltransfer may be as important as sulfonation for the bioactivation of N‐hydroxyaristolactams.

With respect to sulfonation, AL‐I‐NOH and AL‐II‐NOH were mutagenic in *umu* strains expressing human SULT1A enzymes corroborating our earlier findings (Sidorenko et al. 2014). AL‐I‐NOH was genotoxic in SULT1A2‐ and SULT1A1‐expressing bacteria, with only slight effects in the SULT1A3 strain. AL‐II‐NOH was genotoxic in all three *S. thyphimurium*‐expressing human SULT1A1, 1A2, or 1A3. Most importantly, the differential role of SULT1A isoforms in the current study recapitulates previously observed results *in vitro* and in cultured cells (Sidorenko et al. 2014; Hashimoto et al. 2016). Although DCNP reduced the genotoxicities of *N*‐hydroxyaristolactams in SULT1A‐expressing bacteria, supporting the direct role of SULTs in producing their genotoxic intermediates, the *umuC* gene expression was reduced only by half. This finding along with the observed mutagenicity of *N*‐hydroxyaristolactams in the parent strains suggests that there are other mechanisms, which will be discussed somewhere below, involved in the generation of genotoxic species.

### Activation of N‐Hydroxyaristolactams by HK‐2 Cytosols

In the *umu* test, the exact nature of genotoxic species that arises, because of the treatment with test compounds, remains obscure and the potential role of reactive oxygen species in the mutagenicity of the nitroarenes has been discussed above. To explore further whether there are additional bioactivation mechanisms of *N*‐hydroxyaristolactams that yield cyclic nitrenium ions, we selected HK‐2 cells as the source of bioactivation enzymes. These cells originate in the human proximal tubule (Ryan et al. 1994), which is one of the main targets of AA toxicity (Depierreux et al. 1994). Importantly, AA‐I and AL‐I‐NOH are cytotoxic and genotoxic to HK‐2 cells in culture, and these effects are inhibited by pentachlorophenol, SULT1A inhibitor, and to some extent depend on SULT1A1 an SULT1A2 expression (Hashimoto et al. 2016). First, we obtained cytosolic extracts from HK‐2 cells and found that when these fractions are combined with DNA and AL‐I‐NOH, substantial levels of AL‐DNA are formed. Importantly, in this assay, the genotoxicity of N‐hydroxyaristolactams is not mediated by oxidative DNA damage and directly depends on the generation of a cyclic nitrenium ion. Consistent with our previous observations, AL‐I‐NOH reacts only weakly with DNA without the presence of cytosols, and high levels of the compound and long incubations are necessary to be able to detect AL‐DNA, even when using highly sensitive postlabeling method (~5 adducts per 10^9^ nucleotides per 5 μg DNA, corresponding to 0.075 femtomoles of adducts in total).

Surprisingly, when mixtures of AL‐I‐NOH, cytosol, and DNA were fortified by PAPS, it did not promote further AL‐DNA formation. To verify whether this observation was due to the presence of a sufficient amount of PAPS in the cytosolic preparation, we dialyzed HK‐2 cytosols using membranes with the 10 kDa molecular weight cutoff to remove any cofactors present in the original preparation of the cytosols. Following dialysis, HK‐2 fractions showed a drastic loss in their ability to activate AL‐I‐NOH, showing a 500‐fold reduction in activation capacity. This activity could not be restored by the addition of either/or a combination of the following: PAPS, AcCoA, NADPH, NADH, and FAD, suggesting the presence of an unidentified constituent(s) capable of promoting adduct formation. Subsequently, dialysis on 3.5 kDa filter units reduced activation of AL‐I‐NOH only by two‐fold and this was partly due to some loss of protein activity upon defrosting and overnight incubation at 4°C. Since general cofactors have a molecular weight below 1 kDa, these results suggest that there is yet an unknown mechanism(s) that activates *N*‐hydroxyaristolactams into DNA binding species.

### Other Mechanisms Involved in Activation of Hydroxylated Compounds

It remains to be explored, whether a previously unidentified conjugation reaction(s) promotes *N*‐O‐esters formation and AL‐DNA formation, or other changes in *N*‐hydroxyaristolactams facilitated by oxidation or reduction by renal cytosols lead to AL‐DNA. For example, during reduction of AA, the formation of various short‐lived metabolites has been proposed, some of which, for example, oxazinone, may, theoretically, serve as precursors of electrophilic species and AL‐DNA adducts (Priestap et al. 2010; Priestap et al. 2012). Likewise, biotransformation of *N*‐hydroxyaristolactams in cells and extracts may result in various yet to be established metabolites.

With respect to conjugation, in addition to the common esterification reactions, there are other mechanisms that may create electrophilic species (Purohit and Basu 2000). For example, some *N‐O*‐glucuronides exhibit electrophilic properties (Bartsch 1981). *O*‐methylation may potentially create reactive species, but we previously synthesized *N‐O*‐methylester of aristolactam I and it did not bind DNA covalently in contrast to its sulfated species (unpublished data). Effects of phosphorylation, and enzymatic and spontaneous conjugation with glutathione on activities of the *N*‐hydroxyaristolactam remain yet to be explored. Additionally, NATs are not the only enzymes that may be responsible for *O*‐acylation. Some t‐RNA synthesizes were proposed to be involved in activation of certain nitroaromatic compounds in eukaryotes (Tada and Tada 1975).

*In conclusion*, our data shed light on the bioactivation pathways of the aristolochic acids and their proximate carcinogenic species, emphasizing the unusual nature of these compounds, and suggests the existence of a variety of pathways involved in their genotoxicities. Our current research results warrant further investigation of the mechanisms of bioactivation of these important human carcinogens and nephrotoxins.

#### *ACKNOWLEGMENTS*

Authors would like to express their gratitude to Christopher Eyermann for editing the manuscript.

## FUNDING

VS, RB, SA, FJ, and APG were funded by Marsha and Henri Laufers Foundation and Zickler Family Foundation.

## CONFLICT OF INTEREST

The authors declare no conflict of interests.

## AUTHOR'S CONTRIBUTION

Y. Okuno carried out the *umu* assay.

Y. Oda and VS developed the conceptual aspects of the investigation and designed the studies, coordinated the project, analyzed data obtained in *umu* assay, and drafted the manuscript. VS also developed approaches to study the bioactivation of the *N*‐hydroxyaristolactams by cell lysates, conducted experiments involving HK‐2 cytosols, analyzed the results, and prepared the corresponding figures.

RB, SA, and FJ developed the synthesis of and prepared purified AL‐I‐NOH, AL‐II‐NOH, AA‐I, also dA‐AL‐, dG‐AL‐containing oligonucleotides.

FJ and APG aided in study design and in editing the manuscript.

All authors contributed to manuscript writing and editing.

## References

[em22321-bib-0001] Administration FDA. 2001 Aristolochic Acid: Listing of Botanical Ingredients of Concern.[Internet]. [cited 2013 Nov 2013]. Available from: http://www.fda.gov/Food/RecallsOutbreaksEmergencies/SafetyAlertsAdvisories/ucm095283.htm.

[em22321-bib-0002] Alexandrov LB , Jones PH , Wedge DC , Sale JE , Campbell PJ , Nik‐Zainal S , Stratton MR . 2015 Clock‐like mutational processes in human somatic cells. Nat Genet 47(12):1402–1407. 26551669 10.1038/ng.3441 PMC4783858

[em22321-bib-0003] Arlt VM , Meinl W , Florian S , Nagy E , Barta F , Thomann M , Mrizova I , Krais AM , Liu M , Richards M , et al. 2016 Impact of genetic modulation of SULT1A enzymes on DNA adduct formation by aristolochic acids and 3‐nitrobenzanthrone. Arch Toxicol. 91(4):1957–1975. 27557898 10.1007/s00204-016-1808-6 PMC5364269

[em22321-bib-0004] Attaluri S , Bonala RR , Yang IY , Lukin MA , Wen Y , Grollman AP , Moriya M , Iden CR , Johnson F . 2010 DNA adducts of aristolochic acid II: Total synthesis and site‐specific mutagenesis studies in mammalian cells. Nucleic Acids Res 38(1):339–352. 19854934 10.1093/nar/gkp815 PMC2800210

[em22321-bib-0005] Attaluri S , Iden CR , Bonala RR , Johnson F . 2014 Total synthesis of the aristolochic acids, their major metabolites, and related compounds. Chem Res Toxicol 27(7):1236–1242. 24877584 10.1021/tx500122x PMC4216193

[em22321-bib-0006] Bamias G , Boletis J . 2008 Balkan nephropathy: Evolution of our knowledge. Am J Kidney Dis 52(3):606–616. 18725017 10.1053/j.ajkd.2008.05.024 PMC7115735

[em22321-bib-0007] Bartsch H . 1981 Metabolic activation of aromatic amines and azo dyes. IARC Sci Publ 40:13–30. 6799396

[em22321-bib-0008] Boelsterli UA , Ho HK , Zhou S , Leow KY . 2006 Bioactivation and hepatotoxicity of nitroaromatic drugs. Curr Drug Metab 7(7):715–727. 17073576 10.2174/138920006778520606

[em22321-bib-0009] Chen CH , Dickman KG , Moriya M , Zavadil J , Sidorenko VS , Edwards KL , Gnatenko DV , Wu L , Turesky RJ , Wu XR , et al. 2012 Aristolochic acid‐associated urothelial cancer in Taiwan. Proc Natl Acad Sci U S A 109(21):8241–8246. 22493262 10.1073/pnas.1119920109 PMC3361449

[em22321-bib-0010] Chen CJ , Yang YH , Lin MH , Lee CP , Tsan YT , Lai MN , Yang HY , Ho WC , Chen PC , Health Data Analysis in Taiwan Research G . 2018 Herbal medicine containing aristolochic acid and the risk of hepatocellular carcinoma in patients with hepatitis B virus infection. Int J Cancer. 2018. 10.1002/ijc.31544. 29667191

[em22321-bib-0011] Cosyns JP , Jadoul M , Squifflet JP , Wese FX , van Ypersele de Strihou C . 1999 Urothelial lesions in Chinese‐herb nephropathy. Am J Kidney Dis 33(6):1011–1017. 10352187 10.1016/S0272-6386(99)70136-8

[em22321-bib-0012] Dawson WR . 1927 Birthwort: A study of the progress of medicinal botany through twenty‐two centuries. Pharmaceutical J Pharmacist 396‐397:427–430.

[em22321-bib-0013] Depierreux M , Van Damme B , Vanden Houte K , Vanherweghem JL . 1994 Pathologic aspects of a newly described nephropathy related to the prolonged use of Chinese herbs. Am J Kidney Dis 24(2):172–180. 8048421 10.1016/s0272-6386(12)80178-8

[em22321-bib-0014] Dong H , Suzuki N , Torres MC , Bonala RR , Johnson F , Grollman AP , Shibutani S . 2006 Quantitative determination of aristolochic acid‐derived DNA adducts in rats using 32P‐postlabeling/polyacrylamide gel electrophoresis analysis. Drug Metab Dispos 34(7):1122–1127. 16611860 10.1124/dmd.105.008706

[em22321-bib-0015] Fernando RC , Schmeiser HH , Scherf HR , Wiessler M. 1993 Formation and persistence of specific purine DNA adducts by 32P‐postlabelling in target and non‐target organs of rats treated with aristolochic acid I. IARC Sci Publ 124:167–171. 8225480

[em22321-bib-0016] Gillerot G , Jadoul M , Arlt VM , van Ypersele De Strihou C , Schmeiser HH , But PP , Bieler CA , Cosyns JP . 2001 Aristolochic acid nephropathy in a Chinese patient: Time to abandon the term "Chinese herbs nephropathy"? Am J Kidney Dis 38(5):E26. 11684578 10.1053/ajkd.2001.28624

[em22321-bib-0017] Glatt H . 2000 Sulfotransferases in the bioactivation of xenobiotics. Chem Biol Interact 129(1–2):141–170. 11154739 10.1016/s0009-2797(00)00202-7

[em22321-bib-0018] Gold LS , Slone TH . 2003 Aristolochic acid, an herbal carcinogen, sold on the web after FDA alert. N Engl J Med 349(16):1576–1577. 14561805 10.1056/NEJM200310163491619

[em22321-bib-0019] Grollman AP . 2013 Aristolochic acid nephropathy: Harbinger of a global iatrogenic disease. Environ Mol Mutagen 54(1):1–7. 23238808 10.1002/em.21756

[em22321-bib-0020] Grollman AP , Marcus DM . 2016 Global hazards of herbal remedies: Lessons from Aristolochia: The lesson from the health hazards of Aristolochia should lead to more research into the safety and efficacy of medicinal plants. EMBO Rep 17(5):619–625. 27113747 10.15252/embr.201642375 PMC5341512

[em22321-bib-0021] Grollman AP , Shibutani S , Moriya M , Miller F , Wu L , Moll U , Suzuki N , Fernandes A , Rosenquist T , Medverec Z , et al. 2007 Aristolochic acid and the etiology of endemic (Balkan) nephropathy. Proc Natl Acad Sci U S A 104(29):12129–12134. 17620607 10.1073/pnas.0701248104 PMC1913550

[em22321-bib-0022] Hashimoto K , Zaitseva IN , Bonala R , Attaluri S , Ozga K , Iden CR , Johnson F , Moriya M , Grollman AP , Sidorenko VS . 2016 Sulfotransferase‐1A1‐dependent bioactivation of aristolochic acid I and N‐hydroxyaristolactam I in human cells. Carcinogenesis 37(7):647–655. 27207664 10.1093/carcin/bgw045 PMC4936383

[em22321-bib-0023] Hein DW , Doll MA , Rustan TD , Gray K , Feng Y , Ferguson RJ , Grant DM . 1993 Metabolic activation and deactivation of arylamine carcinogens by recombinant human NAT1 and polymorphic NAT2 acetyltransferases. Carcinogenesis 14(8):1633–1638. 8353847 10.1093/carcin/14.8.1633

[em22321-bib-0024] Hoang ML , Chen CH , Sidorenko VS , He J , Dickman KG , Yun BH , Moriya M , Niknafs N , Douville C , Karchin R , et al. 2013 Mutational signature of aristolochic acid exposure as revealed by whole‐exome sequencing. Sci Transl Med 5(197):197ra102. 10.1126/scitranslmed.3006200 PMC3973132 23926200

[em22321-bib-0025] Hoang ML , Chen CH , Chen PC , Roberts NJ , Dickman KG , Yun BH , Turesky RJ , Pu YS , Vogelstein B , Papadopoulos N , et al. 2016 Aristolochic acid in the etiology of renal cell carcinoma. Cancer Epidemiol Biomarkers Prev 25(12):1600–1608. 27555084 10.1158/1055-9965.EPI-16-0219 PMC5533284

[em22321-bib-0026] Hollstein M , Moriya M , Grollman AP , Olivier M . 2013 Analysis of TP53 mutation spectra reveals the fingerprint of the potent environmental carcinogen, aristolochic acid. Mutat Res 753(1):41–49. 23422071 10.1016/j.mrrev.2013.02.003 PMC3689860

[em22321-bib-0027] Hsieh SC , Lin IH , Tseng WL , Lee CH , Wang JD . 2008 Prescription profile of potentially aristolochic acid containing Chinese herbal products: An analysis of National Health Insurance data in Taiwan between 1997 and 2003. Chin Med 3:13. 18945373 10.1186/1749-8546-3-13 PMC2584031

[em22321-bib-0028] Hu SL , Zhang HQ , Chan K , Mei QX . 2004 Studies on the toxicity of *Aristolochia manshuriensis* (Guanmuton). Toxicology 198(1–3):195–201. 15138042 10.1016/j.tox.2004.01.026

[em22321-bib-0029] Jelakovic B , Castells X , Tomic K , Ardin M , Karanovic S , Zavadil J . 2015 Renal cell carcinomas of chronic kidney disease patients harbor the mutational signature of carcinogenic aristolochic acid. Int J Cancer 136(12):2967–2972. 25403517 10.1002/ijc.29338 PMC4720973

[em22321-bib-0030] Kumar V , Poonam PAK , Parmar VS . 2003 Naturally occurring aristolactams, aristolochic acids and dioxoaporphines and their biological activities. Nat Prod Rep 20(6):565–583. 14700200 10.1039/b303648k

[em22321-bib-0031] Maggini V , Menniti‐Ippolito F , Firenzuoli F . 2018 *Aristolochia*, a nephrotoxic herb, still surfs on the web, 15 years later. Intern Emerg Med. 13:811–813. 29498013 10.1007/s11739-018-1813-2

[em22321-bib-0032] Meinl W , Pabel U , Osterloh‐Quiroz M , Hengstler JG , Glatt H . 2006 Human sulphotransferases are involved in the activation of aristolochic acids and are expressed in renal target tissue. Int J Cancer 118(5):1090–1097. 16161050 10.1002/ijc.21480

[em22321-bib-0033] Mengs U . 1988 Tumour induction in mice following exposure to aristolochic acid. Arch Toxicol 61(6):504–505. 3190449 10.1007/BF00293699

[em22321-bib-0034] Mengs U , Lang W , Poch JA . 1982 The carcinogenic action of Aristolochic acid in rats. Arch Toxicol 51(2):107–119.

[em22321-bib-0035] Michl J , Ingrouille MJ , Simmonds MS , Heinrich M . 2014 Naturally occurring aristolochic acid analogues and their toxicities. Nat Prod Rep 31(5):676–693. 24691743 10.1039/c3np70114j

[em22321-bib-0036] Ng AWT , Poon SL , Huang MN , Lim JQ , Boot A , Yu W , Suzuki Y , Thangaraju S , Ng CCY , Tan P , et al. 2017 Aristolochic acids and their derivatives are widely implicated in liver cancers in Taiwan and throughout Asia. Sci Transl Med 9(412):eaan6446. 29046434 10.1126/scitranslmed.aan6446

[em22321-bib-0037] Nortier JL , Martinez MC , Schmeiser HH , Arlt VM , Bieler CA , Petein M , Depierreux MF , De Pauw L , Abramowicz D , Vereerstraeten P , et al. 2000 Urothelial carcinoma associated with the use of a Chinese herb (*Aristolochia fangchi*). N Engl J Med 342(23):1686–1692. 10841870 10.1056/NEJM200006083422301

[em22321-bib-0038] Oda Y , Nakamura S , Oki I , Kato T , Shinagawa H . 1985 Evaluation of the new system (umu‐test) for the detection of environmental mutagens and carcinogens. Mutat Res 147(5):219–229. 3900709 10.1016/0165-1161(85)90062-7

[em22321-bib-0039] Oda Y , Yamazaki H , Watanabe M , Nohmi T , Shimada T . 1993 Highly sensitive umu test system for the detection of mutagenic nitroarenes in salmonella typhimurium NM3009 having high O‐acetyltransferase and nitroreductase activities. Environ Mol Mutagen 21(4):357–364. 8491215 10.1002/em.2850210407

[em22321-bib-0040] Oda Y , Yamazaki H , Watanabe M , Nohmi T , Shimada T . 1995 Development of high sensitive umu test system: Rapid detection of genotoxicity of promutagenic aromatic amines by salmonella typhimurium strain NM2009 possessing high O‐acetyltransferase activity. Mutat Res 334(2):145–156. 7885366 10.1016/0165-1161(95)90005-5

[em22321-bib-0041] Oda Y , Yamazaki H , Shimada T . 1999 Role of human N‐acetyltransferases, NAT1 or NAT2, in genotoxicity of nitroarenes and aromatic amines in salmonella typhimurium NM6001 and NM6002. Carcinogenesis 20(6):1079–1083. 10357791 10.1093/carcin/20.6.1079

[em22321-bib-0042] Oda Y , Zhang Y , Buchinger S , Reifferscheid G , Yang M . 2012 Roles of human sulfotransferases in genotoxicity of carcinogens using genetically engineered umu test strains. Environ Mol Mutagen 53(2):152–164. 22072630 10.1002/em.20696

[em22321-bib-0043] Petljak M , Alexandrov LB . 2016 Understanding mutagenesis through delineation of mutational signatures in human cancer. Carcinogenesis 37(6):531–540. 27207657 10.1093/carcin/bgw055

[em22321-bib-0044] Pfau W , Pool‐Zobel BL , von der Lieth CW , Wiessler M . 1990a The structural basis for the mutagenicity of aristolochic acid. Cancer Lett 55(1):7–11. 2245413 10.1016/0304-3835(90)90058-6

[em22321-bib-0045] Pfau W , Schmeiser HH , Wiessler M . 1990b 32P‐postlabelling analysis of the DNA adducts formed by aristolochic acid I and II. Carcinogenesis 11(9):1627–1633. 2401053 10.1093/carcin/11.9.1627

[em22321-bib-0046] Pfau W , Schmeiser HH , Wiessler M . 1990c Aristolochic acid binds covalently to the exocyclic amino group of purine nucleotides in DNA. Carcinogenesis 11(2):313–319. 2302759 10.1093/carcin/11.2.313

[em22321-bib-0047] Poon SL , Pang ST , McPherson JR , Yu W , Huang KK , Guan P , Weng WH , Siew EY , Liu Y , Heng HL , et al. 2013 Genome‐wide mutational signatures of aristolochic acid and its application as a screening tool. Sci Transl Med 5(197):197ra101. 10.1126/scitranslmed.3006086 23926199

[em22321-bib-0048] Poon SL , Huang MN , Choo Y , McPherson JR , Yu W , Heng HL , Gan A , Myint SS , Siew EY , Ler LD , et al. 2015 Mutation signatures implicate aristolochic acid in bladder cancer development. Genome Med 7(1):38. 26015808 10.1186/s13073-015-0161-3 PMC4443665

[em22321-bib-0049] Priestap HA , de los Santos C , Quirke JM . 2010 Identification of a reduction product of aristolochic acid: Implications for the metabolic activation of carcinogenic aristolochic acid. J Nat Prod 73(12):1979–1986. 21141875 10.1021/np100296y PMC3040066

[em22321-bib-0050] Priestap HA , Barbieri MA , Johnson F . 2012 Aristoxazole analogues. Conversion of 8‐nitro‐1‐naphthoic acid to 2‐methylnaphtho[1,2‐d]oxazole‐9‐carboxylic acid: Comments on the chemical mechanism of formation of DNA adducts by the aristolochic acids. J Nat Prod 75(7):1414–1418. 22746540 10.1021/np300137f

[em22321-bib-0051] Purohit V , Basu AK . 2000 Mutagenicity of nitroaromatic compounds. Chem Res Toxicol 13(8):673–692. 10956054 10.1021/tx000002x

[em22321-bib-0052] Reddy MV , Randerath K . 1986 Nuclease P1‐mediated enhancement of sensitivity of 32P‐postlabeling test for structurally diverse DNA adducts. Carcinogenesis 7(9):1543–1551. 3017601 10.1093/carcin/7.9.1543

[em22321-bib-0053] National Toxicology Program . 2011 NTP 12th report on carcinogens. Rep Carcinog 12:45–49. 21822324

[em22321-bib-0054] Romanov V , Whyard TC , Waltzer WC , Grollman AP , Rosenquist T . 2015 Aristolochic acid‐induced apoptosis and G2 cell cycle arrest depends on ROS generation and MAP kinases activation. Arch Toxicol 89(1):47–56. 24792323 10.1007/s00204-014-1249-z

[em22321-bib-0055] Rosenquist TA , Grollman AP . 2016 Mutational signature of aristolochic acid: Clue to the recognition of a global disease. DNA Repair (Amst) 44:205–211. 27237586 10.1016/j.dnarep.2016.05.027

[em22321-bib-0056] Ryan MJ , Johnson G , Kirk J , Fuerstenberg SM , Zager RA , Torok‐Storb B . 1994 HK‐2: An immortalized proximal tubule epithelial cell line from normal adult human kidney. Kidney Int 45(1):48–57. 8127021 10.1038/ki.1994.6

[em22321-bib-0057] Sato N , Takahashi D , Chen SM , Tsuchiya R , Mukoyama T , Yamagata S , Ogawa M , Yoshida M , Kondo S , Satoh N , et al. 2004 Acute nephrotoxicity of aristolochic acids in mice. J Pharm Pharmacol 56(2):221–229. 15005881 10.1211/0022357023051

[em22321-bib-0058] Scelo G , Riazalhosseini Y , Greger L , Letourneau L , Gonzalez‐Porta M , Wozniak MB , Bourgey M , Harnden P , Egevad L , Jackson SM , et al. 2014 Variation in genomic landscape of clear cell renal cell carcinoma across Europe. Nat Commun 5:5135. 25351205 10.1038/ncomms6135

[em22321-bib-0059] Schmeiser HH , Pool BL , Wiessler M . 1984 Mutagenicity of the two main components of commercially available carcinogenic aristolochic acid in salmonella typhimurium. Cancer Lett 23(1):97–101. 6378360 10.1016/0304-3835(84)90067-3

[em22321-bib-0060] Schmeiser HH , Pool BL , Wiessler M . 1986 Identification and mutagenicity of metabolites of aristolochic acid formed by rat liver. Carcinogenesis 7(1):59–63. 3510750 10.1093/carcin/7.1.59

[em22321-bib-0061] Schmeiser HH , Schoepe KB , Wiessler M . 1988 DNA adduct formation of aristolochic acid I and II in vitro and in vivo. Carcinogenesis 9(2):297–303. 3338114 10.1093/carcin/9.2.297

[em22321-bib-0062] Schmeiser HH , Janssen JW , Lyons J , Scherf HR , Pfau W , Buchmann A , Bartram CR , Wiessler M . 1990 Aristolochic acid activates ras genes in rat tumors at deoxyadenosine residues. Cancer Res 50(17):5464–5469. 2201437

[em22321-bib-0063] Schmeiser HH , Scherf HR , Wiessler M . 1991 Activating mutations at codon 61 of the c‐ha‐ras gene in thin‐tissue sections of tumors induced by aristolochic acid in rats and mice. Cancer Lett 59(2):139–143. 1884371 10.1016/0304-3835(91)90178-k

[em22321-bib-0064] Schmeiser HH , Nortier JL , Singh R , Gamboa da Costa G , Sennesael J , Cassuto‐Viguier E , Ambrosetti D , Rorive S , Pozdzik A , Phillips DH , et al. 2014 Exceptionally long‐term persistence of DNA adducts formed by carcinogenic aristolochic acid I in renal tissue from patients with aristolochic acid nephropathy. Int J Cancer 135(2):502–507. 24921086 10.1002/ijc.28681

[em22321-bib-0065] Seah VM , Wong KP . 1994 2,6‐Dichloro‐4‐nitrophenol (DCNP), an alternate‐substrate inhibitor of phenolsulfotransferase. Biochem Pharmacol 47(10):1743–1749. 8204091 10.1016/0006-2952(94)90301-8

[em22321-bib-0066] Shibutani S , Kim SY , Suzuki N . 2006 32P‐postlabeling DNA damage assays: PAGE, TLC, and HPLC. Methods Mol Biol 314:307–321. 16673890 10.1385/1-59259-973-7:307

[em22321-bib-0067] Shibutani S , Dong H , Suzuki N , Ueda S , Miller F , Grollman AP . 2007 Selective toxicity of aristolochic acids I and II. Drug Metab Dispos 35(7):1217–1222. 17392392 10.1124/dmd.107.014688

[em22321-bib-0068] Sidorenko VS , Yeo JE , Bonala RR , Johnson F , Scharer OD , Grollman AP . 2012 Lack of recognition by global‐genome nucleotide excision repair accounts for the high mutagenicity and persistence of aristolactam‐DNA adducts. Nucleic Acids Res 40(6):2494–2505. 22121226 10.1093/nar/gkr1095 PMC3315299

[em22321-bib-0069] Sidorenko VS , Attaluri S , Zaitseva I , Iden CR , Dickman KG , Johnson F , Grollman AP . 2014 Bioactivation of the human carcinogen aristolochic acid. Carcinogenesis 35(8):1814–1822. 24743514 10.1093/carcin/bgu095 PMC4123648

[em22321-bib-0070] Stiborova M , Frei E , Arlt VM , Schmeiser HH . 2009 The role of biotransformation enzymes in the development of renal injury and urothelial cancer caused by aristolochic acid: Urgent questions and difficult answers. Biomed Pap Med Fac Univ Palacky Olomouc Czech Repub 153(1):5–11. 19365519 10.5507/bp.2009.001

[em22321-bib-0071] Stiborova M , Mares J , Frei E , Arlt VM , Martinek V , Schmeiser HH . 2011 The human carcinogen aristolochic acid i is activated to form DNA adducts by human NAD(P)H:Quinone oxidoreductase without the contribution of acetyltransferases or sulfotransferases. Environ Mol Mutagen 52(6):448–459. 21370283 10.1002/em.20642

[em22321-bib-0072] Stiborova M , Martinek V , Frei E , Arlt VM , Schmeiser HH . 2013 Enzymes metabolizing aristolochic acid and their contribution to the development of aristolochic acid nephropathy and urothelial cancer. Curr Drug Metab 14(6):695–705. 23701164 10.2174/1389200211314060006

[em22321-bib-0073] Tada M , Tada M . 1975 Seryl‐tRNA synthetase and activation of the carcinogen 4‐nitroquinoline‐1‐oxide. Nature 255:510–512. 166317 10.1038/255510a0

[em22321-bib-0074] Tay C. 2019 TGA advises "extreme caution" after detecting cancer‐causing contaminants in Chinese herbal pills. Available from: https://www.nutraingredients-asia.com/Article/2019/2001/2014/TGA-advises-extreme-caution-after-detecting-cancer-causing-contaminants-in-Chinese-herbal-pills#.

[em22321-bib-0075] Totoki Y , Tatsuno K , Covington KR , Ueda H , Creighton CJ , Kato M , Tsuji S , Donehower LA , Slagle BL , Nakamura H , et al. 2014 Trans‐ancestry mutational landscape of hepatocellular carcinoma genomes. Nat Genet 46(12):1267–1273. 25362482 10.1038/ng.3126

[em22321-bib-0076] Turesky RJ , Yun BH , Brennan P , Mates D , Jinga V , Harnden P , Banks RE , Blanche H , Bihoreau MT , Chopard P , et al. 2016 Aristolochic acid exposure in Romania and implications for renal cell carcinoma. Br J Cancer 114(1):76–80. 26657656 10.1038/bjc.2015.402 PMC4716534

[em22321-bib-0077] Vaclavik L , Krynitsky AJ , Rader JI . 2014 Quantification of aristolochic acids I and II in herbal dietary supplements by ultra‐high‐performance liquid chromatography‐multistage fragmentation mass spectrometry. Food Addit Contam Part A Chem Anal Control Expo Risk Assess 31(5):784–791. 24512293 10.1080/19440049.2014.892215

[em22321-bib-0078] Vanherweghem LJ . 1998 Misuse of herbal remedies: The case of an outbreak of terminal renal failure in Belgium (Chinese herbs nephropathy). J Altern Complement Med 4(1):9–13. 9553830 10.1089/acm.1998.4.1-9

[em22321-bib-0079] Vanherweghem JL , Depierreux M , Tielemans C , Abramowicz D , Dratwa M , Jadoul M , Richard C , Vandervelde D , Verbeelen D , Vanhaelen‐Fastre R , Vanhaelen M. 1993 Rapidly progressive interstitial renal fibrosis in young women: Association with slimming regimen including Chinese herbs. Lancet 341(8842):387–391. 8094166 10.1016/0140-6736(93)92984-2

[em22321-bib-0080] World Health Organization . 2008 Some traditional herbal medicines, some mycotoxins, naphthalene and styrene IARC Monographs on the Evaluation of Carcinogenic Risks to Humans. Lyon, France: IARC Press. PMC4781602 12687954

[em22321-bib-0081] Wu TK , Wei CW , Pan YR , Cherng SH , Chang WJ , Wang HF , Yu YL . 2015 Vitamin C attenuates the toxic effect of aristolochic acid on renal tubular cells via decreasing oxidative stressmediated cell death pathways. Mol Med Rep 12(4):6086–6092. 26239057 10.3892/mmr.2015.4167

[em22321-bib-0082] Xing G , Qi X , Chen M , Wu Y , Yao J , Gong L , Nohmi T , Luan Y , Ren J . 2012 Comparison of the mutagenicity of aristolochic acid I and aristolochic acid II in the gpt delta transgenic mouse kidney. Mutat Res 743(1–2):52–58. 22245565 10.1016/j.mrgentox.2011.12.021

[em22321-bib-0083] Yu FY , Wu TS , Chen TW , Liu BH . 2011 Aristolochic acid I induced oxidative DNA damage associated with glutathione depletion and ERK1/2 activation in human cells. Toxicol In Vitro 25(4):810–816. 21300145 10.1016/j.tiv.2011.01.016

[em22321-bib-0084] Zou S , Li J , Zhou H , Frech C , Jiang X , Chu JS , Zhao X , Li Y , Li Q , Wang H , et al. 2014 Mutational landscape of intrahepatic cholangiocarcinoma. Nat Commun 5:5696. 25526346 10.1038/ncomms6696

